# Supplementary material for: Controlling energy levels and Fermi level en route to fully tailored energetics in organic semiconductors
Source: Nat Commun. 2019 Dec 5;10:5538. doi: 10.1038/s41467-019-13563-x (PMC6895164; doi:10.1038/s41467-019-13563-x)
Supplement: Supplementary file 1 — Supplementary Information [file 41467_2019_13563_MOESM1_ESM.pdf]

Supplementary Information for

Controlling energy levels and Fermi level en route to fully tailored energetics in organic semiconductors

Warren et al.

Supplementary Figures

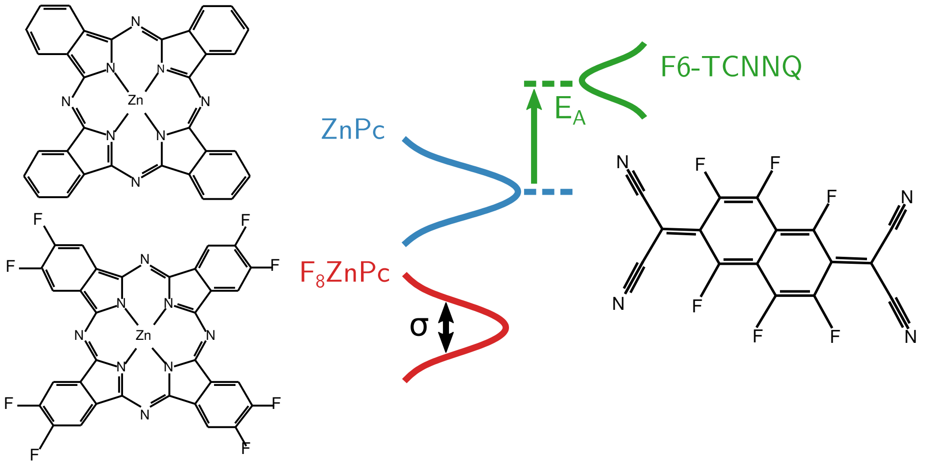

Figure 1: Chemical structures and model sketch. Illustration of the three level model representing the ternary blend alongside the chemical structures of zinc-phthalocyanine (ZnPc), eight-times fluorinated zinc-phthalocyanine (F_8ZnPc) and the dopant, 1,3,4,5,7,8-hexafluorotetracyanonaphthoquinodimethane (F_6-TCNNQ). The density of states (DOS) for each component is assumed to be Gaussian, all with equal standard deviation σ. E_A represents the dopant acceptor level, and is defined as the difference in energy between the centres of the ZnPc DOS and the dopant's DOS. Both E_A and σ are taken as variables in the statistical model.

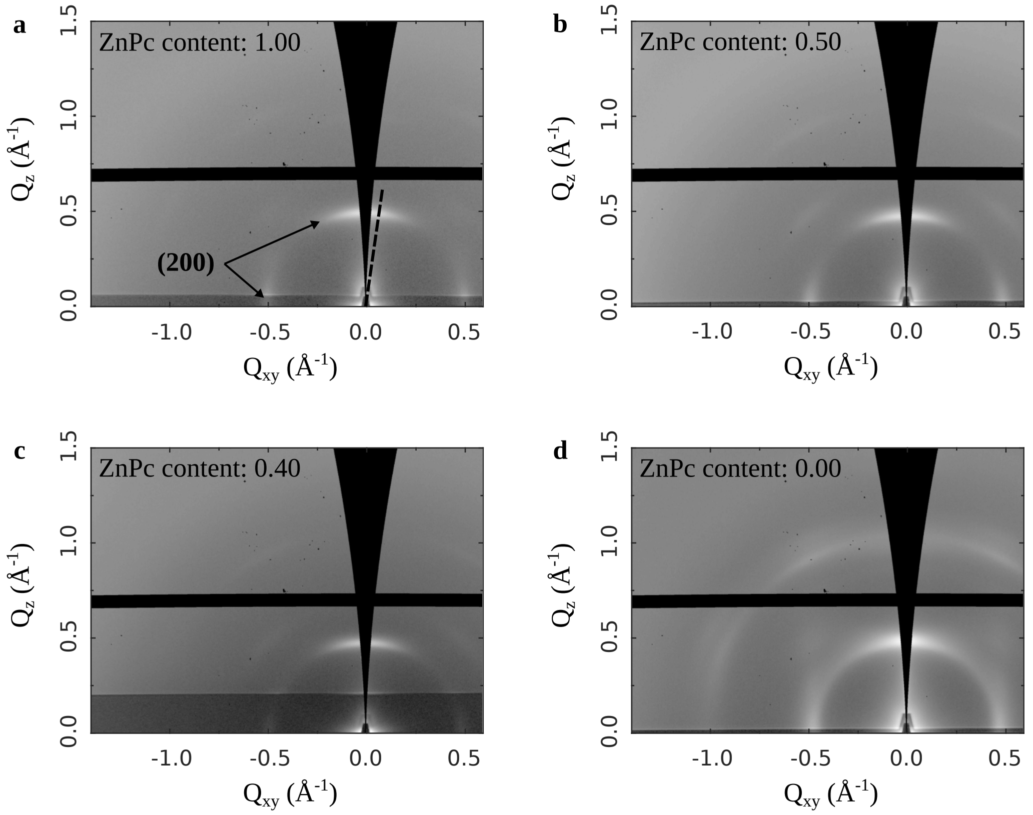

Figure 2: GIWAXS images of p-doped films. GIWAXS 2D reciprocal space maps of films with **(a)** ZnPc content = 1.00, **(b)** ZnPc content = 0.50, **(c)** ZnPc content = 0.40 and **(d)** ZnPc content = 0.00, with doping concentration 0.05 MR, on SiO_2. All films display two preferred orientations either fully in-plane or fully out-of-plane, as seen by the (200) peak, with the out-of-plane orientation being the strongest, indicating a preference for edge-on orientation as compared to face on.

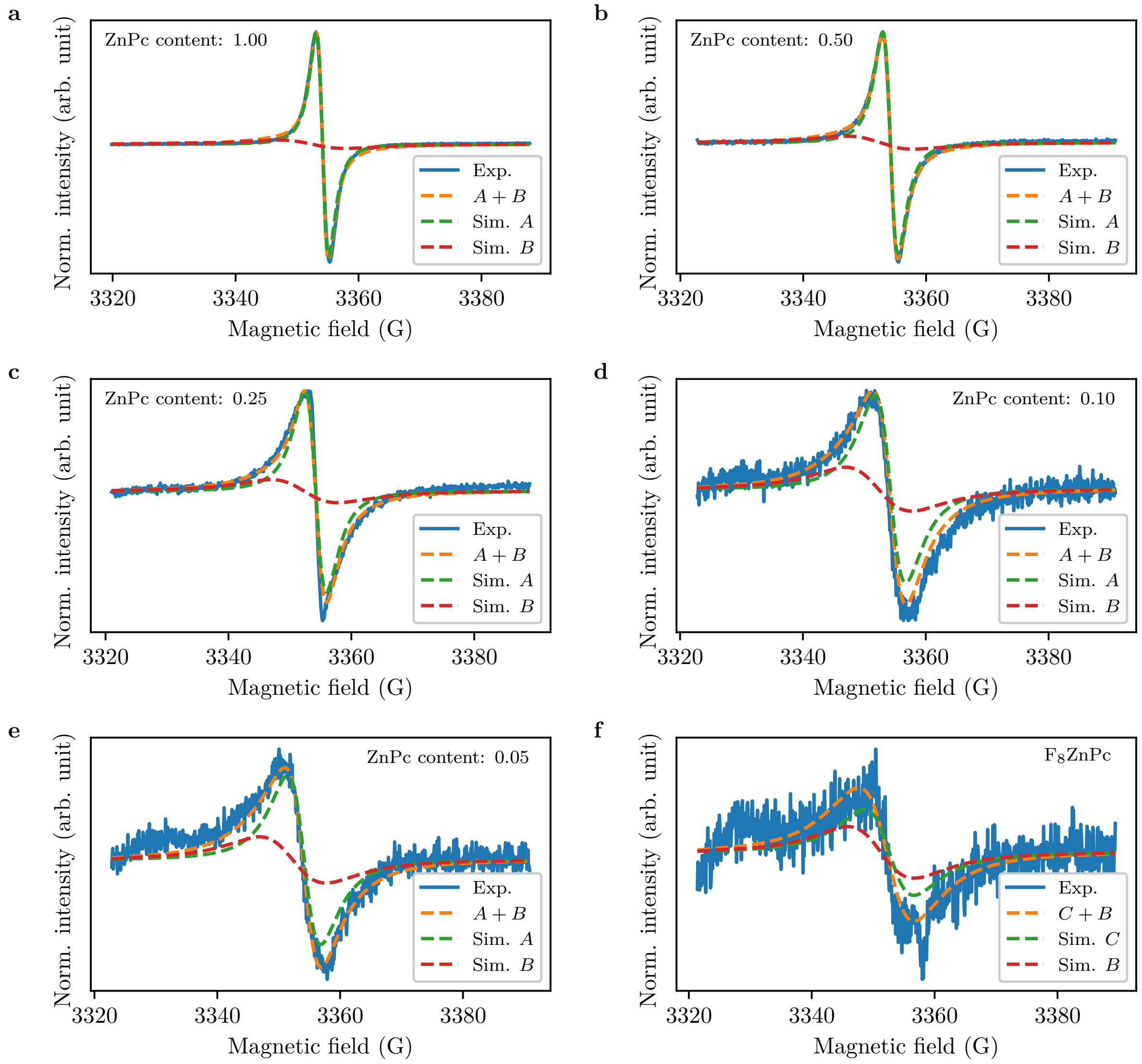

Figure 3: EPR spectra and simulations. Continuous wave EPR spectra for ZnPc:F_8ZnPc blends with weight ratio of ZnPc equal to **(a)** 1.00, **(b)** 0.50, **(c)** 0.25, **(d)** 0.10, **(e)** 0.05 and **(f)** 0.00; all doped with 0.05 MR of F_6-TCNNQ and recorded at room temperature. The dashed orange lines represent the spectral simulations of the EPR spectrum obtained as the sum of two contributions from the ZnPc positive polaron (species A, green dashed line) and the F_6-TCNNQ anion (species B, red dashed line). For the case of F_8ZnPc, the positive polaron is labelled as species C as it has larger g-value.

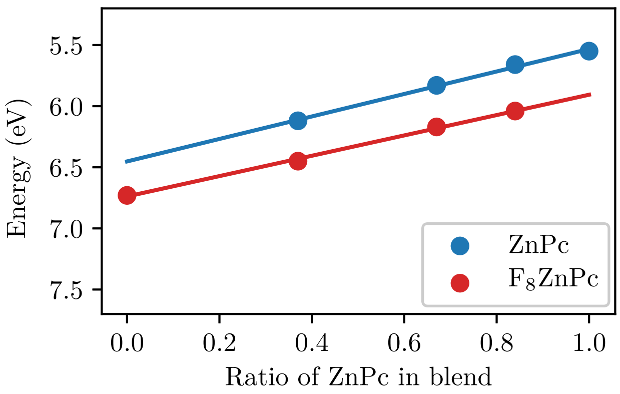

Figure 4: Linear fit of UPS data Linear fit of ultraviolet photoelectron spectroscopy data on blends of ZnPc:F_8ZnPc. The UPS data is from reference [2].

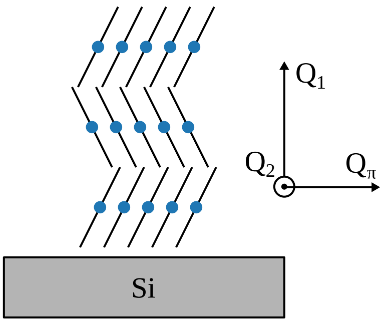

Figure 5: Quadrupole orientation. Schematic diagram showing Q_1, Q_2 and Q_π for edge-on molecules.

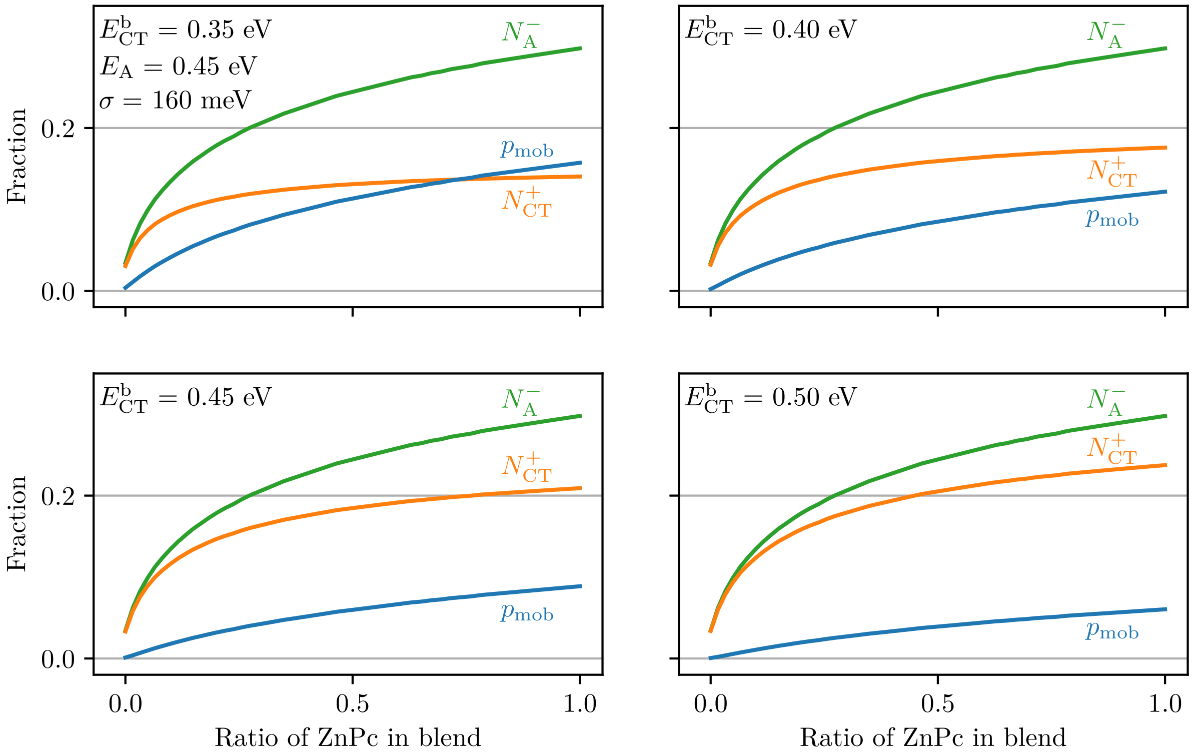

Figure 6: Impact of ICTC binding energy E^{b}_{CT} on charge separation. The effect of varying ICTC binding energy E^{b}_{CT} on the contribution of mobile charges p_{mob}, and charges bound in ICTCs N^{+}_{CT}, in the shifting dopant model. E^{b}_{CT} also shifts, remaining a fixed distance above the ZnPc DOS. The sum of these contributions is equal to the fraction of ionised dopants $p_{mob} + N^{+}_{CT} = N^{-}_{A}$, assuming a negligible contribution from q. The parameters for the simulations are reported inset in the top left panel. The standard deviation of the ICTC states is assumed to be equal to the semiconductor blend $\sigma_{CT} = \sigma$. With increasing E^{b}_{CT}, the proportion of bound charges to mobile charges increases.

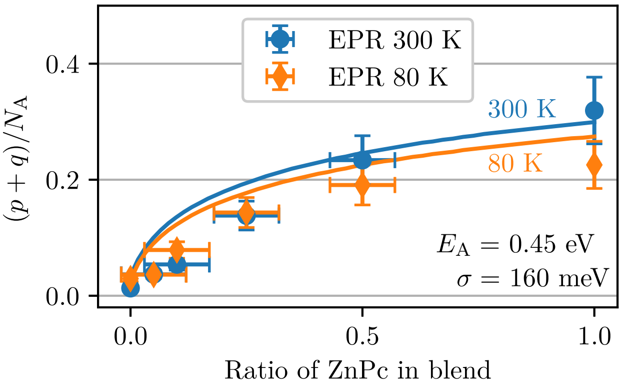

Figure 7: Statistical model comparison to EPR with temperature variation. Comparison of experimentally determined doping efficiency and the statistical model at temperatures $T = 80\,\mathrm{K}$ and $300\,\mathrm{K}$.

Supplementary Tables

ZnPc content	Q [Å^{-1}]	d_{200}-spacing [Å]
1.00	0.488	12.9
0.50	0.472	13.3
0.40	0.470	13.4
0.00	0.479	13.1

Table 1: GIWAXS peak fits and *d*-spacing. Q-values derived from fitted line cuts through 2D GIWAXS images (Supplementary Figure 2) in the near out-of-plane direction through the (200) Bragg peak using a simple sector integration between 7° and 8° from the out-of-plane axial orientation.

ZnPc Content	g_{A}	$linewidth_{\mathrm{A}}$	g_{B}	$linedwidth_{\mathrm{B}}$
1.00	2.0023 ± 0.0005	0.2	2.0033 ± 0.0005	[0.3, 1]
0.50	2.0023 ± 0.0005	0.25	2.0033 ± 0.0005	[0.3, 1]
0.25	2.0023 ± 0.0005	0.35	2.0033 ± 0.0005	[0.3, 1]
0.10	2.0023 ± 0.0005	0.5	2.0034 ± 0.0005	[0.3, 1]
0.05	2.0022 ± 0.0005	0.6	2.0034 ± 0.0005	[0.3, 1]
0.00	2.0032 ± 0.001	0.7	2.0035 ± 0.001	[0.3, 1]

Table 2: EPR g-values and line widths. Table showing the g-values and peak-to-peak line widths of the Lorentzian fits. Species A is attributed to the ZnPc positive polaron and species B is the F_6-TCNNQ anion, except for the case of F_8ZnPc where g_{A} corresponds to the F_8ZnPc positive polaron with a larger g-value (species C).

Molecule	Q_1	Q_2	Q_π
ZnPc	12.35	12.35	-24.69
F_8ZnPc	-14.76	-14.76	29.52
F_6-TCNNQ	-38.91	2.08	36.83

Table 3: Eigenvalues of quadrupole tensor. Values in atomic units, ea_0^2. Q_1 and Q_2 refer to the moments in the in-plane molecular directions and Q_π corresponds to the direction out-of-plane, as shown schematically in Supplementary Figure 5. The B3LYP functional and 6-311g+(d,p) basis set were used in all calculations.

Supplementary Notes

Supplementary Note 1. GIWAXS analysis

Grazing-incidence wide-angle x-ray scattering (GIWAXS) measurements are performed to investigate the crystalline structure of the samples. Supplementary Figures 2(a), 2(b), 2(c) and 2(d) show the 2D reciprocal space maps of the films of mixed ZnPc:F_8ZnPc with the weight ratio of ZnPc in the host-blend equal to 1.0, 0.5, 0.4 and 0, all doped at 0.05 molar ratio (MR). The films display two preferred grain orientations, either fully in-plane or out of plane, as seen by the (200) reflections. The out-of-plane peak is more intense, indicating a preference for edge-on orientation as opposed to face on [1]. All configurations show preferential orientations, which is a precondition for the coherent superposition of the quadrupolar fields of the host molecules [2].

Line cuts are made in the near out-of-plane direction through the (200) Bragg peak using a simple sector integration in DAWN between 7° and 8° from the out-of-plane axial orientation. The fits use a Lorentzian square function of the form:

$$y(x) = A/[1 + (x - pos/a)^2]^2 \tag{1}$$

where $FWHM = 2a(\sqrt{2}-1)$ and $area = \pi a \frac{A}{2}$ and pos is the derived value of Q. The d-spacing values can be found in Supplementary Table 1. These d-spacings are comparable to previously reported values on undoped ZnPc, F_8ZnPc and mixed 1:1. [3]

Supplementary Note 2. Statistical model - calculation details

The occupation of each of the levels is determined by Fermi-Dirac statistics with the Fermi level E_F set by numerically solving the neutrality condition

$$p + q = N_A^-, \tag{2}$$

where p and q are the number of holes, mobile and bound (see Supplementary Note 3) residing on ZnPc and F_8ZnPc, respectively, and N_A^- is the number of ionised dopants. The Fermi-Dirac integrals for the density of charge carriers p and q are given by

$$p, q = \int_{-\infty}^{\infty} g_{p,q}(E)[1 - f(E)]\ dE \tag{3}$$

where $f(E)$ is the Fermi-Dirac distribution and $g_{p,q}(E)$ are the density of states (DOS) for each host material. The DOS are approximated as Gaussian, centred around $E_{p,q}$ with standard deviation σ and normalised to the number of host molecules N_0

$$g_{p,q}(E) = \frac{N_0}{\sqrt{2\pi}\sigma} \exp\left[-\frac{(E - E_{p,q})^2}{2\sigma^2}\right]. \tag{4}$$

The number of ionised dopants N_A^- is determined by an activation from the Fermi level to the dopant acceptor DOS of the form

$$N_A^- = \int_{-\infty}^{\infty} g_A(E, N_A) f(E)\ dE \tag{5}$$

with the dopant DOS $g_A(E)$ also approximated as a Gaussian, centred around E_A with standard deviation σ,

$$g_A(E, N_A) = \frac{N_A}{\sqrt{2\pi}\sigma} \exp\left[-\frac{(E - E_A)^2}{2\sigma^2}\right]. \tag{6}$$

For the fixed dopant level case, the DOS remains static as compared to the shifting host levels. For the shifting dopant case, E_A is redefined relative to the shifting ZnPc DOS, such that it keeps a constant distance E_A from the centre of the ZnPc DOS.

The doping efficiency is defined as the number of ionised dopants divided by the total number of dopants

$$\eta_{dop} = \frac{N_A^-}{N_A} = \frac{p + q}{N_A}. \tag{7}$$

The reference point in energy for all calculations is set such that $E_p \equiv 0\,\text{eV}$ when the ratio of ZnPc in the blend is equal to one. The molar ratio is defined as $MR = N_A/N_0$. The proportion of thermalised free carriers under equilibrium in the host material is assumed negligible ($\leq 10^{-6}$ at 300 K). For all simulations presented in the main text the temperature is kept constant at $T = 300$ K (varied in Supplementary Figure 7).

Supplementary Note 3. Introducing ICTCs

The total number of holes, p and q, on each semiconductor is the sum of mobile carriers, p_{mob} and q_{mob}, and the number bound in ground-state integer-charge transfer complex (ICTCs) between the host and dopant, p_{CT} and q_{CT}:

$$p = p_{\mathrm{mob}} + p_{\mathrm{CT}}, \tag{8}$$

$$q = q_{\mathrm{mob}} + q_{\mathrm{CT}}. \tag{9}$$

The neutrality equation (2) can be rewritten as

$$p_{\mathrm{mob}} + q_{\mathrm{mob}} + N_{\mathrm{CT}}^{+} = N_{\mathrm{A}}^{-}, \tag{10}$$

where $N_{\mathrm{CT}}^{+} = p_{\mathrm{CT}} + q_{\mathrm{CT}}$ is the total number of bound carriers in ICTCs. Ignoring the small fraction of charges residing on F_8ZnPc, the total fraction of charges bound in ICTCs is given by the Fermi-Dirac integral

$$N_{\mathrm{CT}}^{+} = \int_{-\infty}^{\infty} g_{\mathrm{CT}}(E, N_{\mathrm{A}}^{-})[1 - f(E)]\ dE, \tag{11}$$

with the DOS $g_{\mathrm{CT}}(E, N_{\mathrm{A}}^{-})$ approximated as a single Gaussian, centred around $E_{\mathrm{CT}}^{\mathrm{b}}$ (defined with respect to the ZnPc DOS) with standard deviation σ. The effect of introducing ICTCs into the statistical model is plotted in Supplementary Figure 6, with variation in $E_{\mathrm{CT}}^{\mathrm{b}}$ investigated.

Supplementary References

[1] Kim, J. W. et al. High performance organic planar heterojunction solar cells by controlling the molecular orientation. *Current Applied Physics* **13,** 7–11 (2013).

[2] Schwarze, M. et al. Band structure engineering in organic semiconductors. *Science* **352,** 1446–1449 (2016).

[3] Warren, P. R., Hardigree, J. F. M., Lauritzen, A. E., Nelson, J. & Riede, M. Tuning the ambipolar behaviour of organic field effect transistors via band engineering. *AIP Advances* **9,** 035202 (2019).
